# Azacitidine and lenalidomide as an alternative treatment for refractory acute myeloid leukemia: a case report: Azacitidina e lenalidomida como alternativa de tratamento para leucemia mieloide aguda refratária: um relato de caso

**DOI:** 10.1590/1516-3180.2012.6790006

**Published:** 2014-09-19

**Authors:** Juliana Todaro, Patrícia Weinschenker Bollmann, Edna Terezinha Rother, Auro del Giglio

**Affiliations:** I MD. Assistant Professor, Discipline of Hematology and Oncology, Faculdade de Medicina do ABC (FMABC), Santo André, and Hospital Israelita Albert Einstein (HIAE), São Paulo, Brazil.; II MSc. Assistant Professor, Discipline of Hematology and Oncology, Faculdade de Medicina do ABC (FMABC), Santo André, and Hospital Israelita Albert Einstein (HIAE), São Paulo, Brazil.; III Librarian, Institute of Education and Research, Hospital Israelita Albert Einstein (HIAE), São Paulo, Brazil.; IV MD, PhD. Full Professor, Discipline of Hematology and Oncology, Faculdade de Medicina do ABC (FMABC), Santo André, and Hospital Israelita Albert Einstein (HIAE), São Paulo, Brazil.

**Keywords:** Leukemia, myeloid, acute, Drug therapy, Abnormal karyotype, Angiogenesis inhibitors, Azacitidine, Leucemia mieloide aguda, Quimioterapia, Cariótipo anormal, Inibidores da angiogênese, Azacitidina

## Abstract

**CONTEXT:**

Refractory acute myeloid leukemia (AML) is a difficult disease to control with second or third-line chemotherapy regimens. In this report, we describe using azacitidine in combination with lenalidomide as salvage therapy.

**CASE REPORT:**

52-year-old female was diagnosed with refractory AML and high-risk cytogenetics: complex monosomal karyotype consisting of t (3, 3) in association with monosomy 7 and del 5q. Morphological remission associated with maintenance of the cytogenetic abnormality of chromosome 3 and disappearance of the abnormalities relating to chromosomes 5 and 7 was achieved after three cycles of combination therapy with azacitidine and lenalidomide.

**CONCLUSION:**

Azacitidine plus lenalidomide can be a therapeutic option for patients with refractory AML, as illustrated in this case.

## RESUMO

**CONTEXTO:**

A leucemia mieloide aguda (LMA) refratária é considerada doença de difícil controle com regime quimioterápico de segunda ou terceira linha. Neste relato, é descrito o uso de azacitidina em combinação com lenalidomida como esquema de resgate.

**RELATO DE CASO:**

Paciente de 52 anos, do sexo feminino, com o diagnóstico de LMA refratária de alto risco citogenético, apresentava cariótipo complexo e monossômico, com t (3, 3), associado à monosomia do 7 e del 5q. Destaca-se que, após três ciclos da terapia combinada com azacitidina e lenalidomida, houve remissão morfológica, com manutenção da anormalidade citogenética relacionada ao cromossomo 3 e desaparecimento da anormalidade relacionada aos cromossomos 5 e 7.

**CONCLUSÃO:**

Azacitidina e lenalidomida podem ser opção terapêutica para pacientes com LMA refratária, como demonstrado neste caso.

## INTRODUCTION

Refractory acute myeloid leukemia (AML) is a difficult disease to control with conventional second or third-line chemotherapeutic regimens. Most patients die from its complications, and there is not enough time for potentially curative treatment methods such as allogenic bone marrow transplantation to be envisaged.^1^ Therefore, new treatment methods need to be pursued for these patients, so as to allow their disease to be effectively controlled, thereby acting as a bridge to transplantation.

A combination of lenalidomide and azacitidine was recently described for managing AML among elderly patients. The use of these drugs was extrapolated from their action in cases of myelodysplasia.^2^

Here, we describe using azacitidine in combination with lenalidomide as salvage therapy in a 52 years old AML patient with high-risk cytogenetics: complex monosomal karyotype, consisting of t (3, 3) in association with monosomy 7 and del 5q. This patient had been refractory to a combination of Ara C and idarubicin, and to high doses of Ara C.

## CASE REPORT

The patient was a 52-year-old female, who was admitted to our service (Hospital Israelita Albert Einstein, HIAE, São Paulo, Brazil) with fever and was diagnosed with acute leukemia. On admission, she presented hemoglobin 4.8 g/dl (VCM 103.7 fl), leukocytes 45,730/µl with 92% blasts, platelets 64,000/µl and DHL 800 U/L (313-618). A bone marrow aspirate showed that myeloid blast cells accounted for 88.4% of the tissue, and these expressed CD4, CD7, CD11b, CD11c, CD13, CD15, CD33, CD34, CD38, CD71, CD117 and CD123. Cytogenetic analysis demonstrated that there was translocation between the long arms of chromosome 3 and the long arm of chromosome 5 as well as monosomy of chromosome 7 in all the metaphases analyzed (45, XX, t (3, 3) (q21; q26), del (5) (q31q35) - {7} 20). There was no evidence of molecular rearrangements involving FLT3, NPM1 or CBPA.

She received classic induction chemotherapy consisting of idarubicin and cytarabine, without any response, followed by high-dose cytarabine and mitoxantrone (MIDAM)^3^ complicated by soft tissue infection due to fusarium. The anti-fungal treatment involved a combination of voriconazole and caspofungin. On the 14th day of MIDAM, a new bone marrow aspirate showed that blast cells accounted for 70.4% of the material.

We then started her on azacitidine (75 mg/m^2^ on days 1-5) and lenalidomide (25 mg/m^2^ on days 6-19). At the end of the first cycle, she presented morphological remission (blasts in 3.6%). Thus, two further cycles were administered, respecting the interval of 28 days, as a bridge to allogenic unrelated transplantation. Between the second and the third cycle, there was no evidence of blast cells in the bone marrow aspirate.

**Figure 1.**
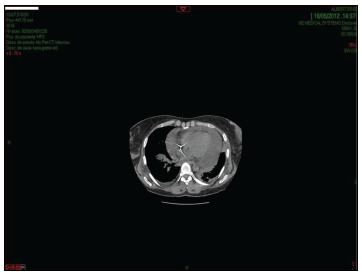
Pericardial effusions after the first cycle with lenalidomide and azacitidine.

She presented pleural and pericardial effusions after the first cycle, which required pericardial percutaneous drainage (Figure 1). Pathology reports from the pericardial biopsy showed only inflammatory changes without evidence of leukemia infiltration (Figure 1). For this reason, in the remaining two cycles, she was given lenalidomide at the reduced dosage of 25 mg/day (days 6-19) and the same dose of azacitidine was maintained.

At the end of the third cycle, she was referred for allogeneic bone marrow transplantation from an unrelated donor (10/10 HLA antigen match) with minimal residual disease (0.51% of the cells were expressing CD7, CD13, CD45 and C34). Her karyotype showed disappearance of monosomy 7 and 5q deletion, but persistence of the translocation involving chromosome 3.

The patient was conditioned using busulfan, fludarabine and thymoglobulin, and received 5 x 10^6^ bone marrow cells, with bone marrow engraftment on day 12. Prophylaxis for graft-versus-host disease (GVHD) was administered, consisting of methotrexate and tacrolimus. Currently, she is doing well on the 90^th^ day after the procedure.

## DISCUSSION

The karyotype of leukemic cells is considered to be a single variable of AML with greater capacity to predict the response to induction chemotherapy and survival. Based on cytogenetic data, AML patients can be categorized into three risk groups: favorable, intermediate and adverse.^4^

Among the adverse prognostic cytogenetic abnormalities are monosomy of chromosomes 5 or 7, deletions on the long arm of chromosome 5, abnormalities on the long arm of chromosome 3 and complex abnormalities (involving at least three distinct aberrations). In fact, in the classification established by the World Health Organization (WHO) in 2008, these abnormalities are grouped in the category AML-related to myelodysplasia.^1^ In our case, besides a complex monosomal karyotype with myelodysplasia-related cytogenetic changes, there was involvement of a nuclear transcription factor EVE-1 (inv (3) (q21; q26.2) or (3, 3) (q21; q26.6)). This transcription factor is related to the mechanism for proliferation and maintenance of hematopoietic cells, and aberrant expression of this factor is implicated in development and progression of high-risk AML.^1^

In patients with cytogenetic findings presenting an adverse prognosis, the current recommendation is to institute consolidation therapy with allogeneic bone marrow transplantation (AloTMO) after evaluation of the response to induction chemotherapy, which is classically performed with a combination of anthracyclines (daunorubicin or idarubicin) and cytarabine (Table 1). This scheme offers a complete response in 60% to 80% of young adults.^1^ Lack of response to this chemotherapy, regardless of cytogenetics, confers a worse outcome. In these cases, prior to AloTMO, rescue therapy based on high-dose cytarabine is chosen in most protocols, with an overall survival of only 20 to 30%.^1^ The combination of lenalidomide and azacitidine, which was proposed by Gotlib for patients older than 60 years who were diagnosed with ‘new’ or secondary AML, achieved a complete response rate of 44%.^2^ We based our decision to start to apply this protocol in this refractory AML patient because of the similarities between her cytogenetics and those of secondary leukemias evolving from myelodysplastic syndrome (MDS), and on experience with elderly patients.^5^^,^^6^^,^^7^ In fact, both lenalidomide and azacitidine present activity in MDS patients and in AML patients with 20-30% bone marrow blasts.^6^^,^^7^^,^^8^ Lenalidomide is an immunomodulatory drug that is active in patients who have been diagnosed with MDS and del 5q,^6^ whereas azacitidine, a hypomethylating agent, can induce hematological responses in MDS with a complex karyotype and monosomy 7.^9^^,^^10^^,^^11^

**Table 1.**
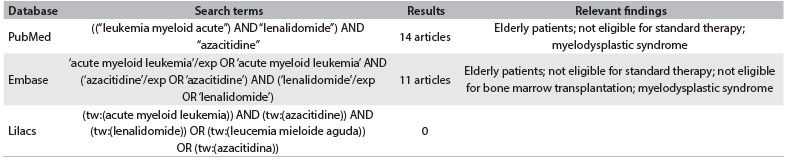
Search strategies conducted in April 2013, and results from the Virtual Health Library, Medline and Lilacs regarding the topics of acute myeloid leukemia and/or lenalidomide and/or azacitidine

We observed that serous effusions were present as a complication. This was not described by Gotlib,^2^ and may have resulted from the higher dose of lenalidomide, of 75 mg/day, which we used in the first cycle.^12^

## CONCLUSION

We conclude that use of the combination of lenalidomide and azacitidine can induce morphological remission in refractory AML and that this should be further studied in larger numbers of patients after failure of conventional chemotherapy.
